# Protocol for massively parallel RNA assay combined with immunoprecipitation for high-throughput analysis of RNA-protein interactions in cells

**DOI:** 10.1016/j.xpro.2026.104624

**Published:** 2026-06-09

**Authors:** Yu Hsuan Lee, John L. Rinn, Taeyoung Hwang

**Affiliations:** 1 Lieber Institute for Brain Development, Johns Hopkins Medical Campus, Baltimore, MD, USA; 2 Department of Biochemistry and BioFrontiers Institute, University of Colorado, Boulder, CO, USA; 3 Department of Neurology, Johns Hopkins University School of Medicine, Baltimore, MD, USA; 4 Solomon H. Snyder Department of Neuroscience, Johns Hopkins University School of Medicine, Baltimore, MD, USA

**Keywords:** Genomics, High Throughput Screening, Molecular Biology

## Abstract

RNA functions are largely mediated through interactions with RNA-binding proteins (RBPs), and defining the molecular principles underlying these interactions is essential for understanding RNA biology. Here, we present a massively parallel RNA assay combined with immunoprecipitation (MPRNA-IP) that enables high-throughput analysis of RNA-protein interactions in cells. We describe detailed steps for oligonucleotide design, cloning, transfection, sequencing, and computational analysis. Together, these procedures allow high-throughput interrogation of RNA sequences to identify sequence and structural elements that contribute to protein binding.

For complete details on the use and execution of this protocol, please refer to Lee et al.^1^

## Highlights

• Instructions for designing and cloning pooled oligonucleotide libraries for MPRNA-IP
• Detailed steps for transfection, immunoprecipitation, and sequencing
• A computational pipeline for identifying RNA elements associated with protein binding

## Abstract (editor highlights)

Publisher’s note: Undertaking any experimental protocol requires adherence to local institutional guidelines for laboratory safety and ethics.

## Graphical abstract

**Figure:**
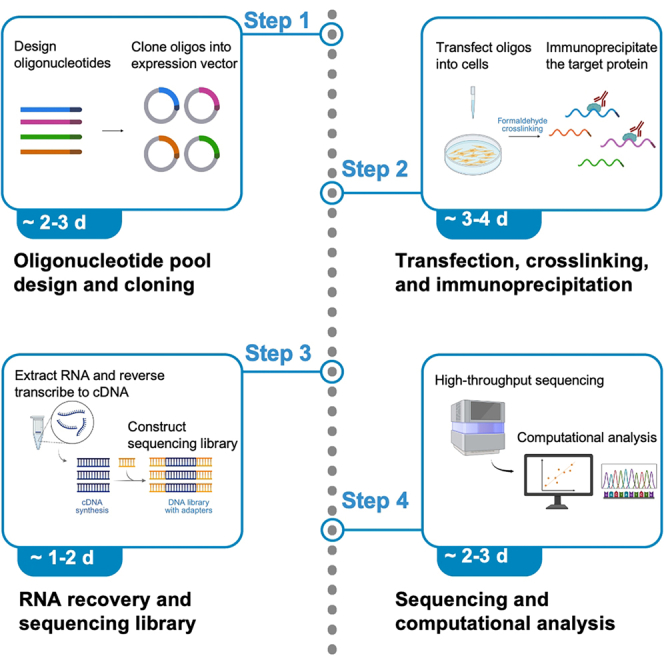

## Before you begin

This protocol details a step-by-step workflow that includes oligonucleotide design for tiling RNA with or without systematic mutations, large-scale oligonucleotide cloning, mammalian cell transfection, formaldehyde crosslinking, immunoprecipitation of RNA-protein complexes, sequencing library preparation, and a complete computational pipeline for downstream analysis. For demonstration of this protocol, we applied it to long noncoding RNA (lncRNA)’s interaction with the RNA-binding protein Pumilio (PUM2) and examined its established binding motif, the Pumilio Response Element (PRE).^2^^,^^3^ A pooled oligonucleotide library tiling five lncRNAs (NORAD, HOTAIR, JPX, XIST, and SRA1) and a control RNA (RNU1-1), with 15 barcodes assigned to each tile, was synthesized comprising 5,835 oligonucleotides. Then, the pool was transfected to HEK293T cells, immunoprecipitated with PUM2, and the PUM2-binding RNA were purified and subjected to high-throughput sequencing. Subsequent computational analysis successfully recovered the PRE motif from the sequencing results, demonstrating the applicability of the approach.

***Note:*** In addition to lncRNA, this protocol can also be adapted to study mRNA–protein interaction studies without changes to the core workflow. For studies focused on post-transcriptional regulation, tiling can be restricted to specific regions of interest, such as the 5′ UTR or 3′ UTR, rather than the full transcript; this targeted approach is more cost-effective and better suited for identifying regulatory elements, as UTRs are well-established sites of RBP interaction. Alternatively, if the goal is to survey the entire transcript without prior assumptions about binding location, full-length tiling can be considered as described here. In this protocol, expressed tiles are driven by a minimal CMV promoter and polyadenylated via an SV40 poly(A) signal, enabling analysis of RNA-protein interactions, particularly in the cytoplasmic context. Also, users should note that tiles are expressed outside their native transcript context and without flanking sequences, which may disrupt long-range RNA interactions that influence structure and RBP binding.

### Innovation

MPRNA is a high-throughput assay that enables the simultaneous investigation of thousands of RNA variants, beyond traditional one-by-one cloning approaches.^4^^,^^5^ We describe a MPRNA workflow specifically designed for probing sequences and structures driving RNA-protein interactions. Our method enhances the MPRNA oligonucleotide design by incorporating multiple molecular barcodes per RNA test sequence, introducing internal technical replicates into the experimental design. This approach increases oligonucleotide representation, reduces the risk of false positives driven by individual barcodes, and improves statistical power in enrichment analyses. We also present a step-by-step computational analysis for comparing Input samples to immunoprecipitated (IP) samples to identify enriched motifs associated with RNA–protein interactions.

### Oligonucleotide pool preparation

**Timing: Variable depending on study**

1. Design oligonucleotide structure.
  a. Define the overall architecture of oligonucleotides. Include an experimental RNA sequence (tile), a unique molecular barcode, and universal adaptor sequences for PCR amplification, arranged in the following order:
       5′ adaptor (16 nt) – tile (157 nt) – molecular barcode (10 nt) – 3′ adaptor (17 nt)
  b. Use the adaptor sequences provided in this protocol:
       5′ adaptor: 5′-ACTGGCCGCTTCACTG-3′
       3′ adaptor: 5′-AGATCGGAAGAGCGTCG-3′
2. Design tile sequences and assign unique molecular barcodes.
  a. Select target RNAs and isoforms. Without specific isoform information, prefer isoforms with the greatest number of exons, or if equivalent, select the longest isoform.
  b. Fragment selected isoforms into overlapping tiles of 157 nt with a 78 nt overlap. If necessary, include tiles covering exons present in alternative isoforms but absent from the selected isoform.
    ***Note:*** Overlapping tiles are designed to provide uniform coverage of annotated lncRNA exonic sequences at a fixed 78 nt resolution, balancing between mapping resolution and the total number of tiles. This resolution is generally sufficient for identifying RNA-binding protein interaction regions, though increased overlap may be desirable for finer mapping. In this protocol, no exclusion criteria are applied based on GC content or predicted secondary structure to avoid introducing systematic gaps and bias against potentially functional regions. Tiles with extreme GC content (<20% or >80%) may be underrepresented due to synthesis and amplification biases.
  c. Assign multiple unique molecular barcodes (10 nt) to each tile.
    **CRITICAL:** Include at least 15 unique barcodes per tile to ensure high reproducibility. This design minimizes loss of tiles during experimental processing, reduces false-positive results driven by single measurement, and increases statistical power in enrichment analyses.
    ***Note:*** Barcodes are computationally generated as random sequences subject to the following design constraints: **(1) Uniqueness**: Barcodes within a pool are designed to be unique to ensure unambiguous assignment of sequencing reads. **(2) Absence of functional sequences**: Barcodes are screened to exclude restriction enzyme recognition sites (e.g., AgeI and NotI) and sequencing adaptor-like sequences to prevent interference with cloning and library preparation. **(3) Sequence composition**: Barcodes with homopolymer runs (≥4 nt) or extreme GC content (<20% or >80%) are excluded to minimize synthesis and sequencing biases. **(4) Minimum edit distance**: A minimum edit distance of 2 between barcodes is recommended to enable detection of sequencing errors.
  d. Assemble the oligonucleotide pool. For this protocol, the pool contains 5,835 oligonucleotides covering five long noncoding RNAs (NORAD, HOTAIR, JPX, XIST, and SRA1) and a control RNA (RNU1-1) (See Table 1 for the number of tiles per RNA), with 15 unique molecular barcodes assigned to each tile. Full oligonucleotide sequences are available in the NCBI GEO repository (see data and code availability).
    ***Note:*** The number of oligonucleotides in MPRNA-IP is primarily limited by synthesis cost, cloning efficiency, and required sequencing depth. Commercial synthesis platforms can generate pools ranging from tens of thousands to hundreds of thousands of sequences, with scale largely constrained by cost. Adequate representation during cloning requires a number of colonies at least 10-fold higher than the pool size, and pilot experiments to assess transformation efficiency are recommended for larger libraries. Additionally, sufficient sequencing depth should be considered, with a recommended minimum of ∼500 sequencing reads per oligonucleotide in Input samples to minimize dropout.

**Table 1:** Summary of the number of tiles per RNA in the oligonucleotide pool

RNA	Isoform	Number of tiles
NORAD	NR_027451	67
HOTAIR	NR_047517	30
JPX	NR_024582	20
XIST	NR_001564	246
SRA1	NR_045586	25
RNU1-1	NR_004430	1
–	–	Total: 389
3. Synthesize the oligonucleotide pool and prepare stock and working solutions.
  ***Note:*** Oligonucleotides can be synthesized through commercial oligo pool services. Upon receipt, store the synthesized oligonucleotide pool according to the vendor’s recommendations. For example, Twist Bioscience recommends a stock concentration of 10 ng/μL for their oligonucleotide pool synthesis service.
  a. Dissolve the Twist Bioscience oligonucleotides in Tris-EDTA (TE) buffer, pH 8.0 (or 10 mM Tris-HCl, pH 8.0) to prepare a stock solution at a concentration of 10 ng/μL.
  b. Dilute the stock oligonucleotide solution to a working concentration of 0.2 ng/μL for the emulsion PCR reaction.

## Key resources table

**Table:** 

REAGENT or RESOURCE	SOURCE	IDENTIFIER
**Antibodies**		

Anti-Pumilio 2 antibody [EPR3813] (Dilution 1:50)	abcam	ab92390

**Chemicals, peptides, and recombinant proteins**		

Acetylated BSA	Invitrogen	AM2614
dNTP Mix	NEB	N0447L
Ultrapure Agarose	Invitrogen	16500100
TAE Buffer (50X)	Quality Biological	351-008-131
LB Broth (Miller)	Sigma-Aldrich	L2542
Ampicillin (100 mg/mL)	Zymo Research	A1001-5
LB Agar Ampicillin Plates (100 μg/mL)	Sigma-Aldrich	L5667
DMEM, high glucose	Gibco	11965092
Fetal Bovine Serum	Corning	35-011-CV
Penicillin-Streptomycin	Gibco	15140122
GlutaMAX Supplement	Gibco	35050061
Trypsin-EDTA (0.5%), no phenol red	Gibco	15400054
Formaldehyde	Sigma-Aldrich	F8775
Glycine	Bio-Rad	1610717
cOmplete Protease Inhibitor (EDTA-free)	Roche	11873580001
N-Lauroylsarcosine	Sigma-Aldrich	L5125
EDTA (0.5 M), pH 8.0	Invitrogen	AM9260G
DTT	Thermo Scientific	R0861
Proteinase K	Invitrogen	AM2546
RNaseOUT	Invitrogen	10777019
Random Primers	Promega	C1181
PBS (10X), pH 7.4	Quality Biological	119-069-131
PBS, pH 7.4	Gibco	10010023
Tris-HCl (1 M), pH 8.0	Quality Biological	351-007-101
Tris-HCl (1 M), pH 7.5	Quality Biological	351-006-101
KCl (2 M)	Invitrogen	AM9640G
SDS (20%, RNase-free)	Invitrogen	AM9820
Triton X-100	Sigma-Aldrich	T8787-50ML
Sodium Deoxycholate	Sigma-Aldrich	D6750-25G
NP-40 Surfact-Amps	Thermo Scientific	28324
Dynabeads Protein G	Invitrogen	10003D
Tween-20	Sigma-Aldrich	P7949
NuPAGE LDS Sample Buffer (4X)	Invitrogen	NP0007
NuPAGE Reducing Agent (10X)	Invitrogen	NP0009
NuPAGE MOPS SDS Running Buffer (20X)	Invitrogen	NP0001
Intercept (TBS) Blocking Buffer	Licor	927-60001
Re-Blot Plus Mild Stripping Solution	Millipore	2502
TRIzol Reagent	Invitrogen	15596026
Chloroform	Sigma-Aldrich	2432-25ML
Nuclease-Free Water (not DEPC-treated)	Thermo Scientific	AM9937
1-Butanol	Thermo Scientific	A3831
Ethyl Alcohol	Sigma-Aldrich	E7023

**Critical commercial assays**		

EURx Micellula DNA Emulsion & Purification Kit	Chimerx	E3600-02
Q5 High-Fidelity DNA Polymerase	NEB	M0491S
AMPure XP Beads	Beckman Coulter	A63881
AgeI-HF	NEB	R3552S
NotI-HF	NEB	R3189S
Quick CIP	NEB	M0525
MinElute Reaction Cleanup Kit	Qiagen	28204
QIAquick Gel Extraction Kit	Qiagen	28706
Quick Ligation Kit	NEB	M2200S
One Shot TOP10 Chemically Competent *E. coli*	Invitrogen	C404003
Plasmid Maxi Kit/Midi Kit	Qiagen	12162/12143
X-tremeGENE HP DNA Transfection Reagent	Roche	6366244001
Opti-MEM I Reduced Serum Medium	Gibco	31985062
RNeasy Plus Mini Kit	Qiagen	74134
DNase I	NEB	M0303S
SuperScript IV Reverse Transcriptase	Invitrogen	18090050
Qubit dsDNA HS Assay Kit	Invitrogen	Q32854
Qubit RNA HS Assay Kit	Invitrogen	Q32855
High Sensitivity DNA Kit	Agilent	50674626
GeneRuler 1 kb DNA Ladder	Thermo Scientific	SM0311
Spectra Multicolor Protein Ladders	Thermo Scientific	26625, 26634
PfuUltra II Fusion HotStart Polymerase	Agilent	600672

**Experimental models: Cell lines**		

HEK293T	ATCC	CRL-3216

**Oligonucleotides**		

Primer: ePCR Forward AATAATACCGGTACTGGCCGCTTCACTG	IDT	Custom order
Primer: ePCR Reverse GAGGCCGCGGCCGCCGACGCTCTTCCGATCT	IDT	Custom order
Oligonucleotide Pool	Twist Bioscience	Custom order
Illumina-compatible sequencing indexes (Table S1)	IDT	Custom order

**Recombinant DNA**		

pMPRNA minCMV vector	Addgene	252518

**Software and algorithms**		

barcodeAligner.py	This paper	Python script
barcodeCounter.R	This paper	R script

**Deposited data**		

Raw FASTQ	NCBI GEO	GSE315146

## Materials and equipment

**Table:** RIPA Buffer for MPRNA-IP

Reagent	Final Concentration	Amount
Tris HCl pH 8.0 (1 M)	50 mM	50 μL
KCl (2 M)	150 mM	75 μL
SDS (20%)	0.1%	5 μL
Triton X-100 (100%)	1%	10 μL
EDTA (0.5 M)	5 mM	10 μL
Sodium deoxycholate (Powder stock made to 10%)	0.5%	50 μL
DTT (1 M)	0.5 mM	0.5 μL
Roche cOmplete Protease Inhibitor Cocktail (20X)	1X	50 μL
RNase OUT (40 U/μL)	100 U/mL	2.5 μL
Nuclease-free water	–	747 μL
Total	–	1 mL

Store buffer without DTT, Protease Inhibitor, and RNaseOUT at 4°C up to 3 months.

**CRITICAL:** Add 0.5 mM DTT, 1X Protease Inhibitor (cocktail), and 100 U/mL RNaseOUT on ice freshly before use.

**Table:** fRIP Buffer for MPRNA-IP

Reagent	Final Concentration	Amount
Tris HCl pH 7.5 (1 M)	25 mM	25 μL
KCl (2 M)	150 mM	75 μL
EDTA (0.5 M)	5 mM	10 μL
NP-40 (100%)	0.5%	5 μL
DTT (1 M)	0.5 mM	0.5 μL
Roche cOmplete Protease Inhibitor Cocktail (20X)	1X	50 μL
RNase OUT (40 U/μL)	100 U/mL	2.5 μL
Nuclease-free water	N/A	832 μL
Total	–	1 mL

Store buffer without DTT, Protease Inhibitor, and RNaseOUT at 4°C up to 3 months.

**CRITICAL:** Add 0.5 mM DTT, 1X Protease Inhibitor (cocktail), and 100 U/mL RNaseOUT on ice freshly before use.

**Table:** 3X Reverse Crosslinking Buffer for MPRNA-IP

Reagent	Final concentration	Amount
PBS (10X)	3X	150 μL
N-Lauroylsarcosine (Powder stock made to 10%)	6%	300 μL
EDTA (0.5 M)	30 mM	30 μL
DTT (1 M)	15 mM	7.5 μL
Nuclease-free water	–	12.5 μL
Total	–	500 μL

Store buffer without DTT at 4°C for 3 months.

**CRITICAL:** Add DTT on ice freshly before use.

**Table:** PBS with Protease Inhibitor (PI)

Reagent	Final concentration	Amount
PBS (1X)	1X	19 mL
Roche cOmplete Protease Inhibitor Cocktail (20X)	1X	1 mL
Total	–	20 mL

Prepare on ice immediately before use.

## Step-by-step method details

### Amplification of oligonucleotide pool by emulsion PCR

**Timing: 3 h**

This step uses EURx Micellula DNA Emulsion & Purification Kit to amplify single-stranded DNA oligonucleotides while adding restriction enzyme (RE) sequences. The ePCR step increases the oligonucleotide length by 26 nts. The ePCR primers have the following structures: (extra base + RE sequence + adapter), with AgeI (ACCGGT) at the 5′ end and NotI (CGCCGGCG) at the 3′ end as the RE sequences. Extra bases are added to ensure efficient RE cleavage during subsequent cloning.

ePCR Forward Primer: 5’ - AATAATACCGGTACTGGCCGCTTCACTG – 3’

   ePCR Reverse Primer: 5’ – GAGGCCGCGGCCGCCGACGCTCTTCCGATCT – 3’

1. Create Oil Surfactant Mixture: Mix the following components for 8 reaction volumes in an appropriate tube in the order indicated in the table at 20-25°C.

***Note:*** This protocol is described to generate a stock of ePCR oligonucleotides using 8 reaction volumes to ensure sufficient material for the subsequent cloning step; however, smaller reaction volumes may be sufficient depending on the pool size. The number of reactions can be adjusted accordingly.

**CRITICAL:** Use wide-bore tips for Emulsion Component 2, as it is viscous.

**Table:** 

	Component	Volume (μL) for 1 Reaction	Volume (μL) for 8 Reactions
i	Emulsion Component 1	220	1760
ii	Emulsion Component 3	60	480
iii	Emulsion Component 2	20	160
	Total volume	300	2400

2. Mix the Oil Surfactant Mixture thoroughly by vortexing and keep it on ice until ready to use.
3. Create PCR Water Phase: Mix PCR samples for 8 reaction volumes in an appropriate tube on ice.

**Table:** 

Component	Final Concentration	Amount
NEB Q5 Buffer (5X)	1X	80 μL
Acetylated-BSA [0.1 ug/μL]	0.01 ug/μL	40 μL
Forward Primer [10 uM]	0.25 uM	10 μL
Reverse Primer [10 uM]	0.25 uM	10 μL
dNTPs Mix [10 mM]	200 uM	8 μL
NEB Q5 polymerase [2,000 U/mL]	0.02 U/μL	4 μL
(0.2 ng/μL)	0.004 ng/μL	8 μL
NEB Q5 high GC enhancer [5X] (optional)	1X	80 μL
Water (Nuclease-free)	N/A	160 μL
Total	N/A	400 μL

***Note:*** A GC enhancer is added to improve PCR efficiency for GC-rich oligonucleotide pools. Although not strictly required, it is recommended for oligonucleotide pools with low sequence complexity and high GC content.

4. Mix the PCR Water Phase by inverting several times and keep it on ice.
5. Create ePCR reactions. For each reaction, combine 300 μL of the Oil Surfactant Mixture and 50 μL of the PCR Water Phase in a 1.5 mL LoBind tube.
6. Vortex the mixture at maximum speed for 5 min in a cold room or at 4°C using a vortexer.
7. Briefly centrifuge the tube, then evenly distribute each ePCR reaction mixture into 3 PCR tubes (approximately 110 μL per PCR tube).
8. Perform following PCR program in a thermal cycler:

**Table:** 

Steps	Temperature	Time	Cycles
Initial Denaturation	94°C	30 s	1
Denaturation	94°C	10 s	25
Annealing	57°C	10 s	
Extension	72°C	10 s	
Final Extension	72°C	2 min	1
Hold	4°C	∞	–

9. Combine the triplicates of each corresponding ePCR reaction from the PCR tubes into a 2 mL LoBind tube.
10. Break the emulsion. For each reaction,
  a. Add 1 mL of butanol to the 2 mL LoBind tube from Step 9 and pipette up and down thoroughly.
  b. Transfer 150 μL of the butanol-containing mixture from the 2 mL LoBind tube back into the first PCR tube.
  c. Pipette up and down thoroughly to dissolve any remaining emulsion.
  d. Repeat Steps 10 b-10c for the second PCR tube.
  e. Repeat Steps 10 b-10c for the third PCR tube.
  f. Transfer the fully dissolved contents from the PCR tubes back into the 2 mL LoBind tube.
11. Vortex the 2 mL LoBind tubes containing ePCR products until the solution becomes transparent.
12. Add 400 μL of Orange-DX buffer to the solution and mix by gentle agitation (e.g., invert by hand or on a rotator for 2 min).
13. Centrifuge for 2 min at maximum speed (e.g., 16,000 × *g*).
14. Remove the upper (yellow-colored) organic phase (it should be approx. 1.2-1.3 mL), leaving a small volume on top of the interphase.
15. Determine the number of spin columns to use.

***Note:*** You can use one column per ePCR reaction or combine 2-4 ePCR duplicates in a single column to increase concentration. In our case, we use one spin column for every 4 ePCR duplicates, resulting in a total of 2 spin columns for 8 ePCR reactions when generating ePCR stock.

16. Apply 40 μL of activation Buffer DX onto each spin-column (do not centrifuge) and keep it at 20-25°C until transferring mixtures to spin-column.
17. Transfer the bottom 2 layers of the mixture (aqueous phase and interphase; max 600 μL) into the spin-column/receiver tube assembly.
18. Centrifuge at 11,000 × *g* for 1 min.
19. Remove spin-column. Discard flow-through, and put the spin-column back on top of the tube.

***Note:*** If the total volume of aqueous phase and interphase exceeds 600 μL, repeat Steps 17-19 using the same spin column.

20. Add 500 μL of Wash DX1 Buffer to spin-column, centrifuge at 11,000 × *g* for 1 min.
21. Remove spin-column. Discard flow-through, and put the spin-column back on top of the tube.
22. Add 650 μL of Wash DX2 Buffer to spin-column, centrifuge at 11,000 × *g* for 1 min.
23. Remove spin-column. Discard flow-through, and put the spin-column back on top of the tube.
24. Centrifuge again at 11,000 × *g* for 2 min to remove traces of Wash DX buffer.
25. Place spin-column into a new 1.5 mL microcentrifuge DNA LoBind tube and add 30 μL of Elution DX buffer into the column.

***Note:*** It is possible to reduce the volume of the eluting buffer below the manufacturer suggested 50 μL (no less than 30 μL) to increase the concentration of DNA. However, recovery of DNA will gradually decrease.

26. Incubate for 2 min at 20-25°C.
27. Centrifuge at 11,000 × *g* for 1 min.
28. Pool all eluted DNA (60 μL) into one tube.
29. ePCR DNA purification (1.6X AMPure XP beads cleanup to remove DNA < 100 base pairs (bp)).
  a. Allow the AMPure XP beads to reach 20–25°C for 30 min.
    **CRITICAL:** Shake the bead reagent thoroughly to resuspend any settled beads. The bead reagent should appear homogeneous before use.
  b. Add 1.6X volume of beads to the eluted ePCR DNA (*Vol. of beads = 1.6 x (reaction vol.)*, 96 μL of beads to 60 μL of eluted ePCR DNA), pipette mixing gently to minimize foaming.
  c. Incubate for 5 min at 20–25°C.
  d. Place the tube on a magnet for 2 min (or longer until the solution turns clear) to separate beads from the solution.
  e. Perform this step while the reaction tube is situated on the magnet: Discard the cleared solution by aspiration without disturbing the beads.
  f. Perform the following steps while the reaction tube is situated on the magnet:
    i. Wash beads by dispensing 200 μL of 70% ethanol into the tube.
    ii. Incubate for 30 s at 20–25°C.
    iii. Aspirate the ethanol out and discard.
  g. Repeat Step (f) for a total of two ethanol washes.
  h. Dry beads for 2–3 min at 20–25°C.
  i. Elute DNA from beads by adding 22 μL of elution buffer (water, TRIS-Acetate (10 mM pH 8.0), or TE Buffer (10 mM Tris-Acetate pH 8.0, 1 mM EDTA)), pipette mixing gently to minimize forming.
  j. Incubate for 2 min at 20–25°C.
  k. Place the tube on a magnet for 2 min (or longer until the solution turns clear) to separate beads from the solution.
  l. Transfer ∼20 μL of the supernatant to a new pre-labeled LoBind-tube (leave ∼2 μL in the tube to avoid bead carryover into the final collection tube).
  m. Repeat the elution Steps (i-l) for a second elution to recover the remaining cleaned pool, and transfer the second ∼20 μL of supernatant to the pre-labeled tube from step (l). The total elution volume is 40 μL.
30. Measure the DNA concentration using a Qubit dsDNA HS Assay Kit.

***Note:*** The expected concentration is ∼45 ng/μL when using the example pool with a total of 8 reactions.

31. Check the DNA size using the Agilent Bioanalyzer High Sensitivity DNA Kit with DNA diluted to 0.5 ng/μL (or other capillary electrophoresis platforms).

**Figure 1:**
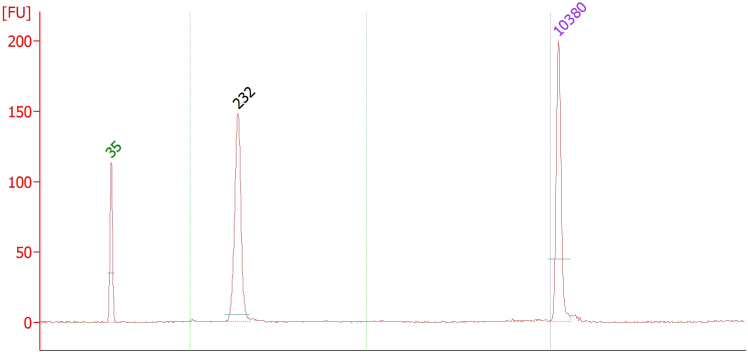
Estimation of oligonucleotide size after ePCR using Bioanalyzer 2100 (X-axis: Base Pairs; Y-axis: Fluorescence Units) The lower (35 bp) and upper (10380 bp) marker peaks serve as internal size references, confirming proper run performance and alignment. The sample peak located between these markers indicates the oligonucleotide size after ePCR of approximately 232 bp. (Step 31).

***Note:*** The expected size of the oligonucleotide pool is 226 bp when using the example pool (based on an oligonucleotide pool of a length of 200 nt). See Figure 1.

***Optional:*** We strongly recommend validating the ePCR product to confirm adequate representation of the oligonucleotide pool. To generate a sequencing library for this purpose, refer to the “Sequencing library generation and sequencing” and “Computational analyses” sections for assessing representation. This step ensures the ePCR product does not miss a significant proportion of oligonucleotides.

**Pause point:** Purified DNA can be stored at −20°C for at least 1 month.

### Oligonucleotide pool cloning into expression vector

**Timing: 2–3 days**

This step inserts the oligonucleotide pool into an expression vector by digesting the oligonucleotides with restriction enzyme AgeI at 5′ end and NotI at 3′ end, followed by ligation into a minCMV expression vector.

32. Insert (ePCR) DNA digestion
  a. Set up the insert (ePCR) DNA digestion reaction as follows:

**Table:** 

Reagent	Amount
ePCR DNA	250 ng (Variable)
NEB CutSmart Buffer (10X)	5 μL
NEB AgeI-HF (20 U/μL)	1 μL
NEB NotI-HF (20 U/μL)	1 μL
Water	to 50 μL
Total	50 μL
  b. Incubate the reaction at 37°C for 5**–**15 min (or incubate at temperature and time ranges recommended for specific RE used).
  c. To stop the enzyme activity, incubate the reaction at 80°C for 20 min.
33. Purification of digested insert (ePCR) DNA:
  a. Purify the digested DNA using MinElute Reaction Cleanup Kit according to the manufacturer’s guide.
  b. Elute the DNA in 10 μL of Buffer EB per column. If multiple columns are used, combine the elute in one tube.
  c. Measure the purified DNA concentration using a NanoDrop Spectrophotometer. The expected concentration is ∼20 ng/μL (based on a DNA input of 250 ng).

**Pause point:** Purified DNA can be stored at −20°C for at least 1 month.

34. Vector digestion
  a. Set up the vector digestion reactions as follows. It is recommended to prepare two additional digestion alongside the vector digestion.

**Table:** 

Reagent	Vector digestion reaction	AgeI single digestion control	NotI single digestion control
Vector	3 ug (variable)	1 ug	1 ug
NEB CutSmart Buffer (10X)	5 μL	5 μL	5 μL
NEB AgeI-HF (20 U/μL)	3 μL	1 μL	N/A
NEB NotI-HF (20 U/μL)	3 μL	N/A	1 μL
Water	to 50 μL	to 50 μL	to 50 μL
Total	50 μL	50 μL	50 μL
    ***Note:*** The yield of digested vector recovered after gel purification is generally 10**–**20% of the input amount. Use an appropriate amount of input vector to ensure sufficient material of subsequent ligation step.
  b. Incubate the reaction at 37°C for 5**–**15 min (or incubate at temperature and time ranges recommended for specific RE used).
  c. To stop the enzyme activity, incubate the reaction at 80°C for 20 min.
35. Gel purification of digested vector.
  a. Prepare 1.2% TAE-agarose gel by dissolving 0.6 g of agarose powder in 50 mL of TAE buffer.
    ***Note:*** The agarose gel percentage can be adjusted according to the length of the vector and the size of the DNA fragment. In this protocol, a 1.2% agarose gel was used for the minCMV vector (2697 bp).
  b. Microwave the agarose mixture for 1 min or until boil. Swirl the mixture to make sure agarose powder is completely dissolved.
  c. Cool the agarose solution on bench for 1**–**2 min.
  d. While the agarose solution is cooling, assemble the gel tray and select an appropriate comb to provide a sufficient number of wells of appropriate size.
    ***Note:*** The recommended amount of DNA to load onto a 1.2% agarose gel is 200 ng per well. If the total DNA amount from a reaction exceeds this recommendation, divide the sample across multiple gel wells to improve band resolution and visualization.
  e. Pour the agarose solution into the assembled tray and allow it to cool and solidify to 20**–**25°C.
  f. Mix 50 μL of each sample (uncut vector, digested vector, AgeI control, and NotI control) with 10 μL of 6X gel loading dye.
  g. Load an appropriate amount of each sample into the gel wells.
  h. Load an appropriate DNA ladder into a well. In this case, a 10 μL of 1kb+ DNA ladder gel is used.
  i. Run the samples for 40 min at 100 V.
    ***Note:*** The time and voltage can be adjusted according to the gel size used. In this case, a 10.5 x 6 cm mini gel is used.
  j. Visualize the DNA by staining the gel with SYBR Green Gel Stain.
    ***Note:*** Cover the gel with the staining solution prepared in TAE buffer at a 1:10,000 dilution (e.g., 10 μL of gel stain dye in 100 mL TAE buffer) and incubate for 30 min on a shaker at 20**–**25°C. Alternatively, premix the agarose solution with appropriate amount of Ethidium Bromide to allow visualization.
  k. Visualize the DNA bands using a UV transilluminator or a blue-light safe imager. Identify the band corresponding to the double-digested vector and excise it from the gel.
    ***Note:*** Run uncut and single-digested controls on the same gel as the double digest. These controls allow identification of the migration positions of uncut and single-cut species, helping to avoid their excision when isolating the fully double-digested vector.
  l. Purify the DNA from the gel slice using QIAquick Gel Extraction Kit according to the manufacturer’s guide.
  m. Elute the DNA in 30 μL of Buffer EB per column. If multiple columns are used, combine the elute in one tube.
  n. Measure the purified DNA concentration using a NanoDrop Spectrophotometer.
    ***Note:*** The expected concentration is 30**–**40 ng/μL (based on the digestion input of 3 ug vector DNA).
    **Pause point:** Purified DNA can be stored at −20°C for at least 1 month.
36. Ligation
  a. Set up the ligation reaction as follows.
    ***Note:*** Digested vector-only control reaction is recommended to be prepared alongside the ligation reaction to access the background from vector self-ligation or incomplete digestion.

**Table:** 

Reagent	Ligation reaction	Digested vector-only control reaction
Digested vector DNA	100 ng (variable)	100 ng (variable)
Digested inserts (4-fold molar excess to vector)	31.2 ng (variable)	N/A
NEB Quick Ligase Buffer (2X)	15 μL	15 μL
NEB T4 Ligase	1 μL	1 μL
Water	to 30 μL	to 30 μL
Total	30 μL	30 μL
    ***Note:*** The insert-to-vector ligation ratio can be optimized based on the length of oligonucleotide pool and the expression vector used. In this protocol, a pool of 200 nt oligonucleotides is ligated into a 2697 bp minCMV vector at a 4:1 molar ratio of insert to vector.
  b. Incubate for 10 min at 20**–**25°C or for 16 h at 16°C.
    **Pause point:** The ligation products can be stored at −20°C for at least 1 month.
37. Transformation
  a. Determine the number of LB-agar plates (containing the appropriate antibiotic for the vector used) required for the oligonucleotide pool. In this protocol, one LB-agar plate is used per 500 oligonucleotides. For example, if the oligonucleotide pool contains 5,000 oligonucleotides, a minimum of 10 LB-agar plates is required to ensure full coverage of the pool.
  b. Determine the number of vials of Invitrogen One Shot TOP10 Chemically Competent Cells required. In this protocol, one vial of competent cells is used per two LB agar plates.
  c. Prepare one additional LB-agar plate and an extra aliquot of competent cells for the digested vector-only control reaction from Step 36 (a).
    ***Note:*** The steps below for transformation are performed according to the Invitrogen One Shot TOP10 Chemically Competent Cells manufacturer’s guide.
  d. Before beginning the transformation:
    i. Equilibrate water bath to 42°C.
    ii. Prewarm appropriate number of selective LB-agar plates (ampicillin) at 37°C for 30 min.
    iii. Prewarm appropriate volume of Stable Outgrowth Medium (S.O.C.) at 37°C for 30 min.
    iv. Pre-heat shaking incubator or Eppendorf thermomixer to 37°C.
  e. Thaw Invitrogen TOP10 Chemically Competent Cells on ice for exactly 5 min.
  f. Add 2**–**3 μL of ligation products into a vial of Invitrogen TOP10 Chemically Competent Cells.
  g. Incubate the vials on ice for 30 min.
  h. Heat shock the cells by placing the vials into a 42°C water bath for 30 s without shaking.
  i. Return the vials back on ice and incubate for 2 min.
  j. Add 250 μL of pre-warmed S.O.C medium into each vial.
  k. Cap the vials and shake the vials in a shaking incubator at 225 rpm, or an Eppendorf thermomixer at 900 rpm, for 1 h at 37°C.
  l. Spread 150 μL of transformation mixture onto a pre-warmed selective LB-agar plate. Allow the mixture to dry for 5 min, then invert the plates. (Total transformation mixture per vial = 300 μL; 150 μL is spread per selective LB-agar plate; 2 LB-agar plates are used per vial of transformation.)
  m. Incubate plates at 37°C for 16 h.
38. Check the number of colonies the next day: successful plates should have >1,000 colonies, and the control plates should have fewer than 5 colonies per plate.
39. Plasmid DNA purification
  a. Add 5 mL of appropriate liquid media (LB + ampicillin) to the successful LB-agar plates.
  b. Gently scrape colonies off the agar plate and into the liquid media with a cell lifter.
  c. Transfer the liquid media to a centrifuge tube (50 mL Falcon tube) on ice.
  d. Add an additional 5 mL of liquid media to the plate.
  e. Gently scrape any remaining colonies off the agar plate and into the liquid media.
  f. Transfer the liquid media to the centrifuge tube (50 mL Falcon tube) on ice, and discard the scraped plate.
  g. Repeat (a-f) for all the plates.
  h. Collect the liquid media and distribute it evenly across the appropriate number of 50 mL centrifuge tubes.
  i. Isolate plasmid DNA using Qiagen Plasmid Maxi Kit (or Midi Kit, depending on the size of oligonucleotide), following the manufacturer’s guide.
  j. Elute DNA in 800 μL of Buffer EB.
  k. Measure plasmid concentration using a NanoDrop Spectrophotometer.

***Note:*** The expected concentration is ∼1000 ng/μL.

***Optional:*** User can confirm adequate representation of the oligonucleotide pool after cloning. To generate a sequencing library for this purpose, refer to the “Sequencing library generation and sequencing” and “Computational analyses” sections for assessing representation. This step ensures the cloning library does not miss a significant proportion of oligonucleotides.

**Pause point:** The purified plasmid oligo pool can be stored at −20°C for 6 months.

### HEK293T cell culture and oligonucleotide pool transfection

**Timing: 2–3 days**

In this step, the oligonucleotide pool plasmid is transfected into HEK293T cells for overexpression of the experimental sequences.

40. Culture HEK293T cells in DMEM, supplemented with 10% FBS, 1% Penicillin-Streptomycin (Pen/Strep), and 1% GlutaMAX Supplement (Gibco).
41. Seed ∼5 million HEK293T cells per 15-cm dish. Allow the cells to grow for 1 day, reaching approximately 50**–**70% confluency before proceeding with transfection.
42. The following transfection steps are performed according to X-tremeGENE HP DNA Transfection Reagent manufacturer’s guidelines. Before beginning the transfection:
  a. Prepare and prewarm cell culture medium without Pen/Strep (DMEM supplemented with 10% FBS and 1% GlutaMAX Supplement (Gibco)) to 15–25°C.
  b. Prewarm X-tremeGENE HP DNA transfection reagent to 15–25°C.
  c. Prewarm Opti-MEM to 15–25°C.
43. Dilute 20 ug of plasmid DNA in 2 mL of Opti-MEM.

***Note:*** The amount of plasmid DNA used for transfection can be optimized. The quantities suggested in this protocol are based on a plasmid size of approximately 3kb to be transfected onto a 50**–**70% confluent HEK293T cells in a 15-cm dish.

44. Briefly vortex the transfection reagent.
45. Add 60 μL of XtremeGENE DNA transfection reagent to each tube of DNA/OptiMEM mixture (3:1 reagent-to-DNA ratio).
46. Gently pipette up and down to mix.
47. Incubate at 20–25°C for 15 min.
48. During the 15 min incubation, rinse cells twice with culture media without Pen/Strep.
49. After aspirating the second rinse, add normal dish volume (∼20 mL for 15 cm dish) of culture media without Pen/Strep.
50. Add transfection complex to the cells in a dropwise manner.
51. Incubate cells in the cell culture incubator for 6 h.
52. Aspirate media, replace it with a normal dish volume of culture media (DMEM, supplemented with 10% FBS, 1% Penicillin-Streptomycin (Pen/Strep), and 1% GlutaMAX Supplement (Gibco)), and return cells to the incubator.
53. Incubate cells for 24–48 h.
54. Once the cells reach 80–100% confluency, proceed to the next step for harvesting.

### Harvesting and formaldehyde crosslinking of RNA-protein complexes

**Timing: 2 h**

This step describes the procedure for harvesting cells and crosslinking RNA-protein complexes with 0.1% formaldehyde.

55. Aspirate the culture medium from the dish.
56. Rinse the cells with cold PBS by adding 20 mL of PBS to the dish. Gently swirl the dish and aspirate the PBS.
57. Harvest the cells by adding 20 mL of cold PBS to the dish, scraping the cells into the PBS, and transferring the cell suspension to a 50 mL conical tube kept on ice.
58. Centrifuge the cells at 500 × *g* for 5 min at 4°C. Discard the supernatant.
59. Resuspend the cell pellet in 2 mL of cold PBS and transfer to a 15 mL conical tube.
60. Add 5.6 μL of 37% formaldehyde to achieve a final concentration of 0.1%.
61. Incubate the tube on a tilt board (or similar tube-rotation device) at 20–25°C for 10 min.
62. Add 100 μL of 2.5 M glycine to achieve a final concentration of 125 mM.
63. Incubate the tube on the tilt board at 20–25°C for 5 min.
64. Centrifuge the cells at 500 × *g* for 5 min at 4°C. Discard the supernatant.
65. Resuspend the cell pellet in 2 mL of cold PBS with Protease Inhibitor.
66. Centrifuge the cells at 500 × *g* for 5 min at 4°C. Discard the supernatant.
67. Resuspend the pellet in 1 mL of cold PBS with Protease Inhibitor and transfer the suspension to a 1.5 mL or 2 mL microcentrifuge tube.
68. Centrifuge the cells at 500 × *g* for 5 min at 4°C. Discard the supernatant.
69. Flash-freeze the pellet in liquid nitrogen or on dry ice.

**Pause point:** The frozen pellets can be stored at −80°C for at least 1 year.

### Cell lysis, sonication for protein extraction, and lysate pre-clearing

**Timing: 1–2 h**

This step describes the procedure for lysing cell pellets, sonicating the lysates to improve protein solubility, and pre-clearing the lysates with beads in preparation for the subsequent immunoprecipitation step.

70. Pre-cool Diagenode Bioruptor Pico to 4°C.
71. Thaw the crosslinked cell pellet and resuspend it in 1 mL of cold RIPA Buffer.
72. Rotate the samples at 4°C for 10 min.
73. Sonicate the samples with Bioruptor Pico:
  a. Add 250 μL of sample lysate to a 1.5 mL Bioruptor tube (4 Bioruptor tubes are required for each pellet containing 1 mL of lysate).
  b. Never leave empty spaces in the tube holder. Fill empty positions with tubes containing the same volume of distilled water.
  c. Select **Easy Mode**: 10 seconds on and 30 seconds off, 3**–**5 cycles at 4°C.

***Note:*** The sonication conditions described here are based on the use of the same lysis buffer, cell density, and other experimental parameters described as this protocol. If the cell density or lysis buffer conditions are changed, optimization of the sonication conditions will be necessary.

74. After sonication is complete, transfer the lysate from Bioruptor tubes to a 1.5 mL microcentrifuge tube.
75. Centrifuge at 15,800 × *g* for 10 min at 4°C.
76. Transfer the supernatant to a new 2 mL tube, and discard the pellet.
77. Add 1 mL of cold fRIP Buffer to bring the total volume to 2 mL.
78. Filter the sample through 0.45-μm filter into a new 2 mL tube.

***Note:*** This step removes large insoluble particles and debris, preventing clogging and ensuring efficient interaction between the lysate, antibody, and magnetic beads in subsequent steps.

79. Prepare Protein G Dynabeads:
  a. Transfer 50 μL of Protein G Dynabeads per sample lysate to a new microcentrifuge tube.
  b. Pull down beads with a magnet, and discard the storage buffer.
  c. Wash the beads twice in 1 mL of cold fRIP Buffer.
  d. Resuspend the beads in original volume of cold fRIP Buffer.
80. Add 50 μL of prepped Protein G Dynabeads to each tube of lysate.
81. Rotate tubes at 4°C for 30 min.
82. Pull down beads by placing tubes on a magnet and transfer the cleared supernatant to a new tube. This is the “pre-cleared lysate”.
83. Take a 25-μL aliquot from each tube of pre-cleared lysate. These aliquots serve as the Input samples.

***Optional:*** Take a second 25-μL aliquot from each tube of pre-cleared lysate as the Input sample for western blot analysis.

84. Flash-freeze pre-cleared lysates and Input aliquots.

**Pause point:** Lysates can be stored at −80°C for at least 1 month.

### Immunoprecipitation of the target protein

**Timing: 4–5 h**

In this step, an antibody specific to the target protein is added to the lysate to immunoprecipitate RNA-protein complexes. Beads are then added to capture the antibody-RNA-protein complexes, followed by washing steps to purify the complexes.

85. Thaw the pre-cleared lysate on ice.
86. Transfer 500**–**1000 μL of pre-cleared lysate (∼5**–**10 million cells) to a 1.5 or 2 mL microcentrifuge tube for each IP condition.
87. Add the appropriate amount of primary antibody to each IP tube according to manufacturer’s guideline.

***Note:*** In this protocol, 2**–**4 ug of Abcam Anti-Pumilio-2 antibody (clone EPR3813) is used per IP sample containing 5**–**10 million cells in 1,000 μL of pre-cleared lysate.

88. Gently vortex the tubes and rotate at 4°C for 2 h (or up to 16 h).
89. Prepare Protein G Dynabeads:
  a. Transfer 50 μL of Protein G Dynabeads per IP condition to a new microcentrifuge tube.
  b. Pull down beads with a magnet, and discard the storage buffer
  c. Wash the beads twice with 1 mL of fRIP Buffer
  d. Resuspend the beads in original volume of cold fRIP Buffer
90. Add 50 μL of Protein G Dynabeads to each IP tube.
91. Rotate at 4°C for 1 h to allow binding of the antibody-RNA-protein complexes to the beads.
92. Pull down the beads with a magnet, and discard the cleared supernatant.
93. Wash the beads as follows:
  a. Resuspend the beads in 1 mL of fRIP Buffer.
  b. Rotate at 4°C for 10 min.
  c. Pull down the beads on a magnet
  d. Discard the cleared supernatant.
94. Repeat Step 93 for 3 additional times (for a total of 4 washes).

***Optional:*** Before aspirating the supernatant from the final wash, transfer 100 μL to a new tube. This 10% aliquot will serve as an IP sample for western blot, while the remaining 90% will be used for RNA isolation.

95. Pull down the beads with a magnet for IP samples (and WB aliquots, if applicable), and discard fRIP Buffer.
96. Store the beads at −80°C.

***Optional:*** Perform western blot analysis to verify successful enrichment of the target proteins. For the expected western blot results, please refer to Supplementary Figure 1B in Lee et al.^1^

**Pause point:** IP Sample beads can be stored at −80°C for at least 1 week.

### RNA isolation and reverse transcription

**Timing: 4–5 h**

This step describes reversing the crosslinks in RNA-protein complexes, isolating RNA using TRIzol combined with the Qiagen RNeasy Plus Mini Kit, and reverse transcribing the isolated RNA into cDNA.

***Note:*** For oligo designs generating RNA smaller than 200 nt, use kits specifically optimized for small RNA isolation and recovery (e.g., miRNeasy).

***Optional:*** Steps 105 – 109 describe DNase treatment after RNA extraction. Alternatively, an on-column DNase digestion can be performed during RNA extraction using the Qiagen RNase-Free DNase Set in combination with RNeasy kit. If this alternative is used, omit Steps 105**–**109. In this case, aliquot 100 ng of RNA in 10 μL and proceed to Step 110.

97. For Input samples (from Step 83), bring volume to 56 μL with nuclease-free water. For IP samples, resuspend beads in 56 μL of nuclease-free water. Transfer all samples into PCR tubes. (For IP samples, transfer everything into the PCR tubes, including the beads.)
98. Add the following to each sample:
  a. 33 μL of 3X Reverse Crosslinking Buffer
  b. 10 μL of 20 mg/mL proteinase K
  c. 1 μL of 40 U/μL RNaseOUT
99. Incubate at 42°C for 1 h.
100. Incubate at 55°C for 1 h.
101. Transfer samples from PCR tubes into new 2 mL microcentrifuge tubes. For IP samples, transfer everything into the new 2 mL tubes, including the beads.
102. RNA isolation with TRIzol and RNeasy Plus Mini Kit.
  a. Add 1 mL of Trizol to each sample and mix briefly by pipetting.
  b. Incubate for 5 min at 20**–**25°C.
  c. Add 200 μL of chloroform to each tube.
  d. Vortex each tube vigorously for ∼15 s.
  e. Incubate for 3 min at 20**–**25°C.
  f. Centrifuge at 12,000 × *g* for 15 min at 4°C.
  g. Transfer the aqueous layers to a new tube.
  h. Add 1 volume (∼500 μL) of 75% ethanol and vortex to mix.
  i. Load 700 μL of each sample at a time onto an RNeasy spin column from the Qiagen RNeasy Plus Mini Kit, each time spinning at 8000 x g for 15 s at 4°C and then discarding the flow-through. Repeat until samples are fully loaded onto the columns.
  j. Add 700 μL Buffer RW1 to the column, spin at 8000 x g for 15 s at 4°C, then discard the flow-through.
  k. Add 500 μL Buffer RPE to the column, spin at 8000 x g for 15 s at 4°C, then discard the flow-through.
  l. Add 500 μL Buffer RPE to the column, spin at 8000 x g for 2 min at 4°C, then discard the flow-through.
  m. Dry columns by spinning at 16,000 x g for 2 min at 4°C.
  n. Transfer each spin column to a new microcentrifuge tube, add 30 μL of RNase-free water to the column, and elute RNA by spinning at 8000 × *g* for 1 min at 4°C.
103. Measure the RNA concentration using a Qubit RNA HS Kit.

***Note:*** The expected concentration for Input samples is approximately 100 ng/μL. For IP samples, RNA may be too diluted to be detected by Qubit.

104. Store RNA samples at −80°C

**Pause point:** RNA samples can be stored at −80°C for at least 1 month.

105. Aliquot 200 ng of RNA from each sample into PCR tubes and adjust the volume to 16 μL with nuclease-free water.

***Note:*** If a given RNA sample is too diluted to obtain 200 ng in 16 μL, just use 16 μL of RNA.

106. Make DNase master mix for each sample:

**Table:** 

Reagent	Amount per sample
NEB DNase I Reaction Buffer (10X)	2 μL
NEB DNase I	2 μL

107. Add 4 μL of master mix to each PCR tube from Step 105.
108. Incubate at 37°C for 30 min.
109. Stop reactions by incubating at 75°C for 10 min.
110. Transfer 10 μL of DNase-treated RNA samples to new PCR tubes.
111. Prepare priming master mix:

**Table:** 

Reagent	Amount per sample
Random hexamers (50 ng/μL)	1 μL
dNTP Mix [10 mM]	1 μL
Water	1 μL

112. Add 3 μL of priming master mix to each tube from Step 110.
113. Incubate at 65°C for 5 minutes, then snap-cool on ice.
114. Prepare RT master mix:

**Table:** 

Reagent	Amount per sample
Invitrogen 5X SuperScript IV Buffer	4 μL
Invitrogen SuperScript IV	1 μL
DTT [100 mM]	1 μL
RNaseOUT [40 U/μL]	1 μL

115. Add 7 μL of RT master mix to each tube (final volume: 20 μL).
116. Run the following program in the thermal cycler:

**Table:** 

Steps	Temperature	Time
Annealing	23°C	10 min
RT	55°C	10 min
Inactivation	80°C	10 min

117. Store cDNA samples at −20°C.

**Figure 2:**
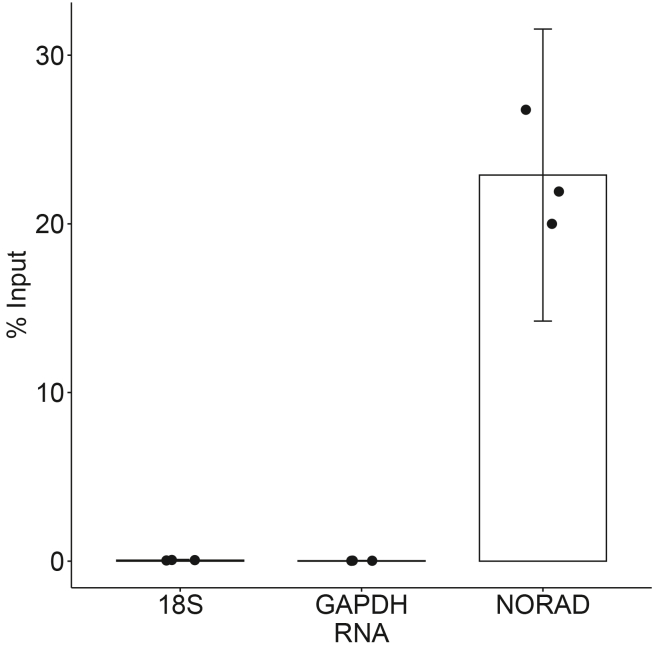
Percent input calculated from qPCR results Using NORAD as a positive control RNA binding to PUM2, along with the negative RNA genes 18S and GAPDH, qPCR analysis shows a high percent input for NORAD in the PUM2 immunoprecipitation (>20%), whereas 18S and GAPDH show minimal enrichment (∼0%). Each dot represents a biological replicate (*n* = 3). Error bars indicate 95% confidence interval. (Optional step after Step 117).

***Optional:*** The cDNA generated in this step can be used to perform a qPCR experiment to assess RNA recovery following immunoprecipitation and to confirm successful pulldown of the target protein. See Figure 2.

**Pause point:** cDNA samples can be stored at −20°C for at least 1 month.

### Sequencing library generation and sequencing

**Timing: 3 h for library generation, sequencing time may be variable depending on library size**

This step generates libraries by attaching Illumina-compatible sequencing adapters to DNA fragments and adding index sequences by PCR. Use different indexes if multiple libraries are generated and multiplexed. See Table S1 for sequencing library primers with indexes.

118. Determine the amount of DNA: ePCR oligo product (1 ng), plasmid library (50 ng), fRIP cDNA (10 μL)
119. Set up the PCR reaction as follows:

**Table:** 

Reagent	Amount
DNA	Variable (1 ng ePCR product, 50 ng plasmid library, 10 μL fRIP cDNA)
10X Pfultra Buffer	5 μL
Pfultra (2.5 U/μL)	1 μL
dNTP Mix (10 mM)	2.5 μL
Primer Forward Index (2 uM)	2.5 μL
Primer Reverse Index (2 uM)	2.5 μL
Water	To 50 μL

120. Run the PCR reaction as follows:

**Table:** (ePCR product: 12 cycles, plasmid library: 18 cycles, fRIP cDNA: 24 cycles)

Steps	Temperature	Time	Cycles
Initial Denaturation	95°C	2 min	1
Denaturation	95°C	30 s	ePCR Product: 12 Plasmid pool: 18 fRIP cDNA: 24
Annealing	55°C	30 s	
Extension	72°C	30 s	
Final Extension	72°C	10 min	1
Hold	4°C	∞	–

121. Library purification: 0.6X Ampure XP beads cleanup to retain DNA<300 bp
  a. Allow the AMPure XP beads to reach 20**–**25°C for 30 min.
    **CRITICAL:** Shake the bead reagent thoroughly to resuspend any settled beads. The bead reagent should appear homogeneous before use.
  b. Add 0.6X volume of beads to the DNA (*Vol. of beads =0.6 x (reaction vol.)*, 30 μL of beads to 50 μL of PCR reaction volume), pipette mixing gently to minimize foaming.
  c. Incubate for 5 min at 20**–**25°C.
  d. Place the tube on a magnet for 2 min (or longer until the solution turns clear) to separate beads from the solution.
  e. Transfer the supernatant to a new PCR tube. Be careful not to carry over any beads during the transfer and note the volume transferred.
122. Library purification: 1.6X Ampure XP beads cleanup to remove DNA<100 bp
  a. Add 1.6X volume of beads to the supernatant transferred from above step (*Vol. of beads = 1.6 x (supernatant vol.)*, 112 μL of beads to 70 μL of supernatant), pipette mix gently to minimize foaming.
  b. Incubate for 5 min at 20-25°C.
  c. Place the tube on a magnet for 2 min (or longer until the solution turns clear) to separate beads from the solution.
  d. Perform this step while the reaction tube is situated on the magnet: Discard the cleared solution by aspiration without disturbing the beads.
  e. Perform this step while the reaction tube is situated on the magnet:
    i. Wash beads by dispensing 200 μL of 70% ethanol into the tube.
    ii. Incubate for 30 s at 20**–**25°C.
    iii. Aspirate the ethanol out and discard.
  f. Repeat the above step for a total of two ethanol washes.
  g. Dry beads for 2**–**3 min at 20**–**25°C.
  h. Elute DNA from beads by adding 22 μL of water, pipette mixing gently to minimize forming.
  i. Incubate for 2 min at 20**–**25°C.
  j. Place the tube on a magnet for 2 min (or longer until the solution turns clear) to separate beads from the solution.
  k. Transfer 40 μL of the supernatant to a new PCR tube.
123. 1.0X Ampure XP beads cleanup
  a. Add 1X volume (40 μL) of beads to the elute from above step, pipette mix gently to minimize foaming.
  b. Incubate for 5 min at 20**–**25°C.
  c. Place the tube on a magnet for 2 min (or longer until the solution turns clear) to separate beads from the solution.
  d. Perform this step while the reaction tube is situated on the magnet: Discard the cleared solution by aspiration without disturbing the beads.
  e. Perform this step while the reaction tube is situated on the magnet:
    i. Wash beads by dispensing 200 μL of 70% ethanol into the tube.
    ii. Incubate for 30 s at 20**–**25°C.
    iii. Aspirate the ethanol out and discard.
  f. Repeat the above step for a total of two ethanol washes.
  g. Dry beads for 2**–**3 min at 20**–**25°C.
  h. Elute DNA from beads by adding 16 μL of water, pipette mixing gently to minimize forming.
  i. Incubate for 2 min at 20**–**25°C.
  j. Place the tube on a magnet for 2 min (or longer until the solution turns clear) to separate beads from the solution.
  k. Transfer 15 μL of the supernatant to a new pre-labeled PCR tube.
124. Measure the DNA concentration using a Qubit dsDNA HS Assay Kit.

***Note:*** The expected concentration is 3**–**10 ng/μL for Input samples and 0.5–3 ng/μL for IP samples.

125. Check the size of the DNA using Agilent Bioanalyzer High Sensitivity DNA Kit with diluted 0.5 ng/μL DNA.

**Figure 3:**
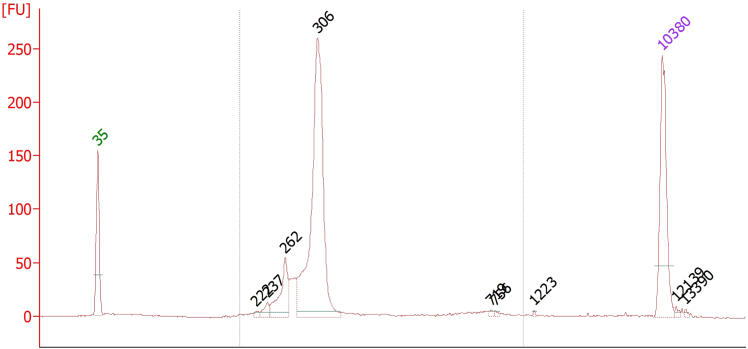
Estimation of sequencing library size of Input samples using Bioanalyzer 2100 (X-axis: Base Pairs; Y-axis: Fluorescence Units) The lower (35 bp) and upper (10380 bp) marker peaks serve as internal size references, confirming proper run performance and alignment. The sample peak located between these markers indicates a library size of Input samples of approximately 306 bp. (Step 125).

**Figure 4:**
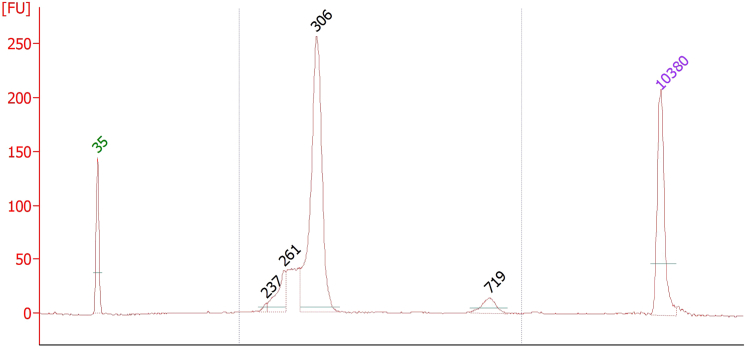
Estimation of sequencing library size of IP samples using Bioanalyzer 2100 (X-axis: Base Pairs; Y-axis: Fluorescence Units) The lower (35 bp) and upper (10380 bp) marker peaks serve as internal size references, confirming proper run performance and alignment. The sample peak located between these markers indicates a library size of IP samples of approximately 306 bp. (Step 125).

***Note:*** The expected size of the library is ∼305 bp. See Figures 3 and 4 for Input and IP samples, respectively.

**Pause point:** Sequencing libraries can be stored in −20°C for at least 1 month.

126. Sequence library on Illumina NGS platforms.

***Note:*** Perform single-end sequencing with a read length longer than the barcode. This protocol uses a 100-cycle kit. Generate at least 500 sequencing reads per oligo to minimize missing oligos. Refer to the supplementary file for sequencing sample index information.

### Computational analyses

**Timing: Variable depending on sequencing size and computational resources**

In this step, sequencing reads are first mapped to the barcodes on the designed oligonucleotides and counted for each oligonucleotide. After oligonucleotides with low counts (Reads Per Million < 1) across all replicates are removed, the counts of remaining oligonucleotides are summed for each tile. The resulting number of sequencing reads assigned to each tile is then compared between Input and IP samples using a linear regression model implemented in the R package DESeq2.^6^ Here, we assume that 3 biological replicates (3 sets of Inputs and IP samples) of fRIP, as well as the ePCR product and the cloning library, were sequenced, generating the total 8 sequencing outputs.

***Note:*** This analysis uses UNIX commands and R scripts. Reference files for *OLIGO_POOL.fasta* (experimental or tile sequences with barcodes), and *TILES.fasta* (experimental or tile sequences only, without barcodes) are required for this analysis. Ensure that all required reference files and sequencing files of all samples (including replicates) are in the same directory.

127. Map sequencing reads to barcodes for all samples. For every zipped FASTQ file (for example, SAMPLE.fastq.gz), run the following command.

zcat SAMPLE.fastq.gz | \

./barcodeAligner.py \

-i stdin \

-o SAMPLE_mapped.txt \

-p OLIGO_POOL.fasta \

-b 10

***Note:*** Sequencing reads from the zipped FASTQ file (SAMPLE.fastq.gz) are decompressed and aligned to the barcodes of oligonucleotide pool using barcodeAligner.py. Reads were piped directly into the program via standard input (-i stdin), and the aligned output (-o) was written to SAMPLE_mapped.txt using the oligonucleotide reference file (-p OLIGO_POOL.fasta) and barcode length (-b) specifies the barcode length of 10 nt used in this oligonucleotide pool design.

128. Construct a count matrix of mapped sequencing reads with tiles as rows and samples as columns. Here, we included 6 fRIP samples (3 pairs of Input and IP), as well as ePCR and Maxiprep samples.

Rscript barcodeCounter.R \

-n ePCR,Maxiprep,R1_IN,R1_IP,R2_IN,R2_IP,R3_IN,R3_IP \

-i ePCR.mapped.txt,Maxiprep.mapped.txt,\

R1_IN.mapped.txt,R1_IP.mapped.txt,\

R2_IN.mapped.txt,R2_IP.mapped.txt,\

R3_IN.mapped.txt,R3_IP.mapped.txt \

-p OLIGO_POOL.fasta \

-t 2,2,2,2,2,2,2,2 \

-o TestPool_ePCR_Maxiprep

***Note:*** barcodeCounter.R can take multiple samples that use the same oligonucleotide pool design: Provide sample names (-n), corresponding mapped files (-i) generated in the previous step, and mismatch thresholds (-t) as comma-separated lists in the same order of samples. When sequencing reads are longer than the barcode length, the sequencing reads with up to two mismatches are counted (-t 2). All samples are processed using the same oligonucleotide pool design file (-p OLIGO_POOL.fasta). The output matrix is saved as a comma-separated text file with the name of the specified prefix (-o TestPool_ePCR_Maxiprep). In this example: IN represents Input sample and IP indicates the corresponding immunoprecipitation (IP) samples of a fRIP experiment. The result file name is “TestPool_ePCR_Maxiprep_count.csv”.

129. Open R and load the required packages. If necessary, install the packages.

if (!requireNamespace("dplyr", quietly = TRUE)) {

 install.packages("dplyr")

}

if (!requireNamespace("reshape2", quietly = TRUE)) {

 install.packages("reshape2")

}

if (!requireNamespace("edgeR", quietly = TRUE)) {

 install.packages("edgeR")

}

if (!requireNamespace("Biostrings", quietly = TRUE)) {

 install.packages("Biostrings")

}

if (!requireNamespace("Biostrings", quietly = TRUE)) {

 install.packages("ggplot2")

}

library(dplyr)

library(reshape2)

library(edgeR)

library(Biostrings)

library(ggplot2)

130. Load count data.

# load oligo count matrix

count.mat = read.csv("TestPool_ePCR_Maxiprep_count.csv", header=T, row.names = 1) %>% as.matrix()

# generate a sample sheet that has sample information

sampleSheet = data.frame(sampleID = colnames(count.mat), repl=c("ePCR", "Maxi", rep(c("Rep1", "Rep2", "Rep3"), each=2)), pulldown = c(NA, NA, rep(c("Input", "IP"), 3)))

# calculate total number of counts for every sample

sampleSheet$countedNum = colSums(count.mat)

131. Organize data and set parameters for analysis.

# flat count data as a data.frame

oligo.count = reshape2::melt(count.mat, varnames = c("oligoID", "sampleID"), value.name = "raw")

oligo.count = mutate(oligo.count, tileID = gsub("_[ˆ_]+$", "", oligoID), barcodeID = gsub("ˆ.∗_", "", oligoID))

oligo.count = left_join(oligo.count, sampleSheet[, c("sampleID", "repl", "pulldown")], by="sampleID")

# calculate RPM (Reads Per Million reads) of oligo

oligo.count$rpm <- oligo.count$raw / (sampleSheet$countedNum[match(oligo.count$sampleID, sampleSheet$sampleID)]/10^∧^6)

# set the parameters

TILE_NUM = length(unique(oligo.count$tileID))

BARCODE_NUM = 15

REPLICATE_NUM = 3

132. Check the representation of oligonucleotides in each sample.

# missing oligo and tile numbers

oligo.count.zero = subset(oligo.count, raw==0)

temp = reshape2::dcast(oligo.count.zero, sampleID∼., value.var = "oligoID")

sampleSheet$mOligoNum <- temp$"."[match(sampleSheet$sampleID, temp$sampleID)]

sampleSheet$mOligoRate <- sampleSheet$mOligoNum / (TILE_NUM∗BARCODE_NUM)

temp = reshape2::dcast(oligo.count.zero, sampleID∼., value.var = "tileID", fun.aggregate = function(x) length(unique(x)))

sampleSheet$mTileNum <- temp$"."[match(sampleSheet$sampleID, temp$sampleID)]

sampleSheet$mTileRate <- sampleSheet$mTileNum / TILE_NUM

print(sampleSheet)

# make a plot of distribution of oligos

p = ggplot(oligo.count, aes(log10(rpm+1))) + facet_wrap(∼sampleID)

p = p + geom_density()

p = p + xlab("Log10 (RPM+1)") + ylab("Density")

p = p + ggtitle("RPM of oligos")

p

# save the plot

ggsave(

 filename = "RPM_of_oligos.pdf",

 plot = p,

 width = 10,

 height = 6,

 units = "in"

)

***Note:*** Check the number of missing oligos and tiles in the samples. Also check the distribution of oligo counts. Unimodal curves are expected for all samples except IP samples.

133. Pool barcodes for each tile after filtering oligonucleotides with RPM<1.

# if necessary, keep the relevant fRIP samples only

oligo.count = subset(oligo.count, sampleID %in% c("R1_IN", "R1_IP", "R2_IN", "R2_IP", "R3_IN", "R3_IP"))

sampleSheet = subset(sampleSheet, sampleID %in% c("R1_IN", "R1_IP", "R2_IN", "R2_IP", "R3_IN", "R3_IP"))

# Define functions for filtering and pooling

oligoFilter <- function(oligo.count, inputFilterBy="rpm", inputTh=0, ipFilterBy="rpm", ipTh=0) {

# this function filters oligonucleotides based on either "raw" or "rpm" values.

oligo.count$oligoID <- paste(oligo.count$tileID, oligo.count$barcodeID, sep="_")

 
 
# Input filter

 
temp <- subset(oligo.count, pulldown=="Input" & get(inputFilterBy)>=inputTh)

 
temp <- table(temp$oligoID)

 
temp <- names(temp)[temp==REPLICATE_NUM] # oligoIDs should be expressed in all the replicates.

oligo.count <- subset(oligo.count, oligoID %in% temp)

 
# IP filter

 
temp <- subset(oligo.count, pulldown=="IP" & get(ipFilterBy)>=ipTh)

 
temp <- table(temp$oligoID)

 
temp <- names(temp)[temp==REPLICATE_NUM] # oligoIDs should be expressed in all the replicates.

 
oligo.count <- subset(oligo.count, oligoID %in% temp)

cat("Proportion of oligos after filtering: ", length(unique(oligo.count$oligoID))/(TILE_NUM∗BARCODE_NUM), "\n")

 
oligo.count$oligoID <- NULL

 
return(oligo.count)

}

# barcode pooling function

countPooler <- function(oligo.count) {

 
oligo.pooled <- dcast(oligo.count, tileID∼sampleID, value.var="raw", fun.aggregate = sum)

 
rownames(oligo.pooled) <- oligo.pooled$tileID

 
pooled.count <- data.matrix(oligo.pooled[,-1]) # Output as a matrix

 
cat("Proportion of tiles after filtering and pooling: ", nrow(pooled.count)/TILE_NUM, "\n")

 
return(pooled.count)

}

# Perform filtering and pooling

pooled.count <- oligoFilter(oligo.count, inputFilterBy="rpm", inputTh=1, ipFilterBy="rpm", ipTh=1) %>% countPooler()

134. Identify the tiles enriched in IP relative to IN samples.

# Normalization

edgeR.obj <- DGEList(pooled.count)

edgeR.obj <- calcNormFactors(edgeR.obj, method="TMM")

# Voom to use count data for linear modeling

pooled.model <- model.matrix(data= sampleSheet, ∼0 + pulldown + repl)

y <- voom(edgeR.obj, pooled.model, plot = T)

# Linear model fit by limma

fit <- lmFit(y, pooled.model)

# Linear model contrast

contr <- makeContrasts(pulldownIP - pulldownInput, levels = colnames(coef(fit)))

out <- contrasts.fit(fit, contr)

# Empirical Bayes smoothing of standard errors (shrinks standard errors that are much larger or smaller than those from other genes towards the average standard error)

out <- eBayes(out)

# Results

tile.res <- topTable(out, sort.by = "P", n = Inf) %>% tibble::rownames_to_column("tileID")

# hist(tile.res$P.Value) # Optional: check the distribution of p-values.

# Enriched tiles

tile.res$enriched <- "Insig"

tile.res$enriched[which(tile.res$adj.P.Val<0.01 & tile.res$logFC>0)] <- "Enriched"

tile.res$enriched[which(tile.res$adj.P.Val<0.01 & tile.res$logFC<0)] <- "Depleted"

table(tile.res$enriched)

***Note:*** A tile is labeled as Enriched if its fold change (on a log_2_ scale) in IP relative to Input is greater than 0 and false discovery rate is less than 0.01.

135. Generate a ranked list of tiles.

# Read a fasta file of tile sequences.

tile.seq = readDNAStringSet("TILES.fasta")

# Write a fasta file for the ordered tiles.

temp <- tile.seq[tile.res$tileID[order(tile.res$t, decreasing = T)]]

writeXStringSet(temp, "PUM2-MPRNA_ranked.fasta")

136. Identify sequence motif using DRIMust^7^
  a. Go to DRIMust at https://drimust.technion.ac.il/
  b. Input sequence
    i. Select Ranked list
    ii. Upload SAMPLE_ranked.fasta
    iii. Select search mode as single-strand
  c. Search parameter
    i. Set motif range: Min. length = 4 and Max. length = 20
    ii. Set statistical significance threshold as 10^∧^-2
  d. Submit

**Figure 5:**
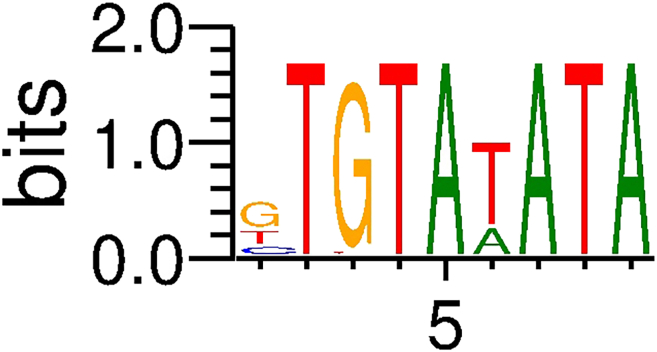
Motif results produced by DRIMust Using the oligonucleotide pool described in this protocol and following the outlined steps, the sequence motif associated with PUM2 was identified. (Step 135).

***Note:*** As this protocol quantifies tile enrichment relative to each other, rank-based motif analysis tools such as DRIMust are preferred. However, users may also use other popular motif analysis tools, such as MEME.^8^ As an example of the motif analysis produced by DRIMust, see Figure 5. In this analysis, we successfully identified the Pumilio-binding element, the Pumilio Response Element (PRE), characterized by the consensus sequence UGUA[A/C/U]AUA.^2^

## Expected outcomes

This protocol enables systematic identification of RNA sequence motifs associated with a protein of interest. After expressing a large number of short RNA fragments (tiles) in cells, NGS sequencing is performed on immunoprecipitation (IP) and total lysates (Input) samples, generating a matrix of sequencing read counts for each tile across Input and IP samples. Tiles that are reproducibly enriched in IP relative to Input can be identified, providing a quantitative measure of RNA-protein interactions.

The resulting motifs provide insight into the sequence features that drive RNA-protein binding and can inform downstream functional studies, such as mutational analysis or validation in cellular assays. Overall, the expected outputs of this protocol include a matrix of sequencing read counts for all tiles in the designed oligonucleotide pool, a set of reproducibly enriched sequences, and the derived sequence motifs associated with the protein of interest.

For additional data generated using this protocol, please refer to Lee et al.^1^

## Limitations

A limitation of this protocol is the requirement for transfection of a pooled oligonucleotide library, which restricts its application to cell types that are amenable to efficient transfection and may not fully recapitulate endogenous regulatory contexts. Additionally, formaldehyde crosslinking can introduce bias by capturing indirect RNA–protein interactions and perturbing native binding dynamics. Variability in crosslinking efficiency and reversal may further influence RNA recovery and quantitative accuracy. These factors should be carefully considered when interpreting MPRNA-IP results.

## Troubleshooting

### Problem 1

The concentration of emulsion PCR products is lower than expected (Step 30).

### Potential solution

Adding a GC enhancer to the PCR reaction may improve PCR efficiency for oligonucleotide pools with GC-rich sequences.

### Problem 2

Few colonies are observed after ligation (Step 38).

### Potential solution

Increasing the insert-to-vector molar ratio may improve ligation efficiency.

### Problem 3

Transfection efficiency is low (Steps 41**–**54).

### Potential solution

Optimize the amount of plasmid DNA used for transfection or adjust the transfection reagent–to–DNA ratio.

### Problem 4

Immunoprecipitation of the protein of interest is unsuccessful (Steps 86**–**96).

### Potential solution

• Optimize the sonication step to improve protein solubility and accessibility for immunoprecipitation.
• Adjust the amount of antibody used to ensure sufficient binding.
• If endogenous expression of the target protein is low, consider overexpressing the protein alongside the oligonucleotide pool or using a different cell type with higher expression levels.

### Problem 5

The yield of RNA after RNA isolation is lower than expected (Step 103).

### Potential solution

For low RNA concentrations from Input samples.

• Ensure RNase inhibitors are properly added to all buffers throughout the experiment.
• Use sufficient cell lysate; consider increasing the number of cells.

For low RNA concentrations from immunoprecipitation samples:

• This is generally expected, as RNA yields from IP are typically low before amplification and may not indicate a problem.

### Problem 6

The size distribution of oligonucleotides or libraries shows multiple peaks (Step 125).

### Potential solution

• Check oligonucleotides and vector sequences for potential nonspecific binding or repeat sequences.
• Reduce the number of PCR cycles to prevent overamplification and the formation of undesired products.

## Resource availability

### Lead contact

Further information and requests for resources and reagents should be directed to and will be fulfilled by the lead contact, Taeyoung Hwang (taeyoung.hwang.@libd.org).

### Technical contact

Technical questions on executing this protocol should be directed to and will be answered by the technical contact, Yu Hsuan Lee (yuhsuan.lee@libd.org).

### Materials availability

Plasmids related to this study will be available through Addgene plasmid no. 252518, but standard mammalian expression vectors with CMV promoters can be used as alternatives.

### Data and code availability

The accession number for the sequencing and the design files reported in this paper is NCBI GEO: GSE315146. All codes and the tile information file (TILES.fasta) have been deposited to Mendeley Data: https://doi.org/10.5281/zenodo.19680142.

## Acknowledgments

We thank Yong Kyu Lee and Dr. Joo Heon Shin of the sequencing core at the Lieber Institute for Brain Development. This work was supported by 10.13039/100000002NIH grant R00GM137072 to T.H. The graphical abstract was created in BioRender. Lee, Y. H. (2026) https://BioRender.com/0w37yk5.

## Author contributions

T.H. conceived the study. Y.H.L. performed the experiments and T.H. analyzed the data. J.L.R. consulted the overall study. Y.H.L. and T.H. wrote the paper.

## Declaration of interests

The authors declare no competing interests.

## Declaration of generative AI and AI-assisted technologies in the writing process

During the preparation of this work, the authors used AI agents (ChatGPT and Claude) in order for language editing and improving clarity. After using this tool/service, the authors reviewed and edited the content as needed and take full responsibility for the content of the published article.

## Supplemental information

Table S1. Sequencing primers and indexes, related to Step 119
